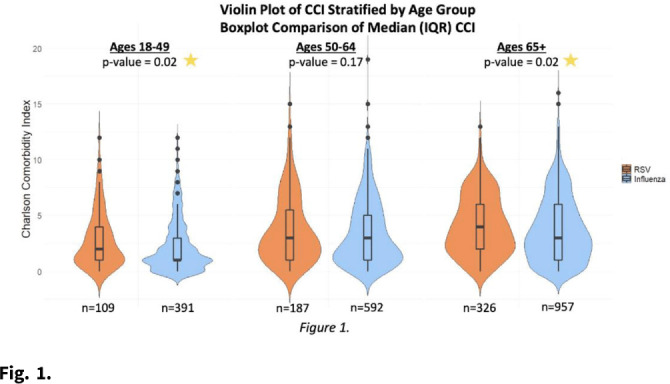# Relevance of RSV in hospitalized adults and the need for continued testing

**DOI:** 10.1017/ash.2022.176

**Published:** 2022-05-16

**Authors:** Katherine Miller, Arnold Monto, H. Keipp Talbot, Manjusha Gaglani, Tresa McNeal, Fernanda Silveira, Richard Zimmerman, Donald Middleton, Shekhar Ghamande, Kempapura Murthy, Lindsay Kim, Jill Ferdinands, Manish Patel, Emily Martin

## Abstract

**Background:** RSV is underrecognized in hospitalized adults. A better understanding of RSV in this population could help prioritize targeted viral-testing resources. Hospitalization and in-hospital outcomes are widely accepted as markers of clinical severity with respect to acute respiratory illness (ARI). We compared characteristics and clinical outcomes between adults hospitalized with ARI from October 2016 through May 2019. **Methods:** All hospitalized adults (≥ 18 years) who met a standardized case definition of ARI were prospectively enrolled across 3 respiratory seasons from 9 hospitals participating in the US Hospitalized Adult Influenza Vaccine Effectiveness Network (HAIVEN). Demographic data were collected during enrollment interviews, and electronic medical records (EMRs) were reviewed to extract comorbidity data. Throat and nasal swabs collected at enrollment were tested for ARI pathogens using real-time PCR assays at respective HAIVEN research laboratory sites. Characteristics and clinical outcomes of participants were compared using χ^2^ or nonparametric tests where appropriate. Multivariable logistic regression models were used to test associations between infection status, characteristics, and clinical outcomes, adjusting for age, sex, race, Charlson comorbidity index (CCI), body mass index (BMI), site, season, and days to admission. **Results:** In total, 10,311 adults were included, 22.3% (n = 2,300) were aged 18–49 years, 33.2% (n = 3,423) were aged 50–64 years, and 44.5% (n = 4,588) were aged ≥65 years. Moreover, 6% of adults tested positive for RSV (n = 622), 18.8% positive for influenza (n = 1,940), and 75.1% negative for both (n = 7,749). Obesity and age ≥65 years were significantly associated with RSV detection when compared with participants negative for both RSV and influenza. Patients aged 18–49 years and ≥65 years with RSV had significantly higher median CCI scores compared to patients with influenza (Fig. 1.). The proportion of adults with CHF or COPD was significantly (p-value **Conclusions:** Severe RSV illness may differ from severe influenza illness, and those infected with RSV may have different characteristics than those infected with influenza. Hospitalized adults with RSV infection were more likely to have underlying cardiopulmonary comorbidities and higher CCI scores as well as experience an extended length of hospital stay and need for mechanical ventilation. These data highlight the importance of retaining testing for RSV in older adults hospitalized with ARI.

**Funding:** None

**Disclosures:** None

**Figure:**